# Sustaining surveillance as an intervention during the COVID-19 pandemic in Cabo Verde and implications for malaria elimination

**DOI:** 10.3389/fimmu.2022.956864

**Published:** 2022-10-06

**Authors:** Adilson DePina, Helga Barros, Amanda Tiffany, Gillian Stresman

**Affiliations:** ^1^ Programa de Eliminação do Paludismo, CCS-SIDA, Ministério da Saúde, Praia, Cabo Verde; ^2^ Ecole Doctorale Sciences de la Vie, de la Santé et de l’Environnement - Université Cheikh Anta Diop, Dakar, Senegal; ^3^ Unidade de Estatística, Delegacia de Saúde da Praia, Praia, Cabo Verde; ^4^ Global Malaria Programme, World Health Organization, Geneva, Switzerland; ^5^ Department of Infection Biology, Faculty of Infectious and Tropical Diseases, London School of Hygiene & Tropical Medicine, London, United Kingdom; ^6^ Epidemiology Department, College of Public Health, University of South Florida, Tampa, FL, United States

**Keywords:** malaria elimination, COVID-19, surveillance, public health and social measures, Cabo Verde

## Abstract

Cabo Verde reported the first case of COVID-19 on March 19, 2020. Containment measures were quickly implemented and over 80,000 COVID-19 tests were performed in 2020 with 11,840 confirmed infections (2% of the population) and 154 deaths. In a setting where the last locally acquired malaria case was reported in January 2018, any interruptions to malaria care-seeking have the potential for infections to go untreated and transmission re-establishing. This work aims to determine whether there was any change in the number of people seeking care or being tested for malaria and, using an interrupted time series analysis, identify if any change was associated with implemented COVID-19 measures. Routinely collected surveillance data for outpatient visits, testing for malaria and COVID-19 were aggregated by month for each health facility (outpatient and malaria) or by municipality (COVID-19) from 2017 through 2020. The timeline of COVID-19 measures was generated based on when and where they were implemented. Results show that there was a marked shift in care-seeking in Cabo Verde. Overall, the mean number of observed outpatient visits decreased from 2,057 visits per month during 2017-2019 to 1,088 in 2020, an estimated 28% reduction. However, malaria testing rates per 1,000 outpatient visits after the pandemic began increased by 8% compared to expected trends. Results suggest that the pandemic impacted care-seeking but led to a non-significant increase in testing for malaria per 1,000 outpatient visits. With the cessation of international travel, the risk of imported infections seeding new transmission declined suggesting the risk of undetected transmission was low. It is important for countries to understand their specific malaria risks and vulnerabilities in order to ensure that any progress towards the interruption of malaria transmission can be sustained.

## Introduction

The COVID-19 pandemic caused by the severe acute respiratory coronavirus 2 (SARS-CoV-2) prompted governments in many countries to implement measures to help contain the spread of the virus (1). These measures include restricting travel internationally and domestically, mask mandates, stay at home orders or lockdown and mandatory quarantine periods, amongst others (2). Some studies show that the measures imposed helped reduce the incidence of COVID-19 by reducing population mobility and potential exposure to the virus (3). However, additional information on the direct and indirect impact of COVID-19 measures on attitude and behavior changes on health care utilization for non-COVID-19 related infections, both short and long-term, is needed to ensure that disease surveillance trends can be interpreted appropriately (4).

One pandemic related impact may be a change in care-seeking. Recent studies have shown that the COVID-19 pandemic has impacted care-seeking for various health issues. When comparing pre- and post-pandemic periods, a reduction in care-seeking was associated with reduced prescriptions for antibiotics in outpatient departments was reported in Canada (5), reductions in hospital presentations for self-poisoning was reported in Sri Lanka (6), decreased visits for other respiratory disorders was reported in China (7), and lower attendance at antenatal and vaccination clinics was reported in Uganda (8). Conversely, the pandemic has also been associated with an increase in care-seeking; a recent study in China found an increase in care-seeking for conditions related to the nervous system (7).

The ongoing COVID-19 pandemic may also impact the probability that a patient is tested if they seek care for their illness (e.g., malaria, HIV, Tuberculosis, other infectious and chronic diseases). Factors that may influence the likelihood of testing include symptom presentation, prevalence of the illness, availability of testing or treatment supplies, the ability of the health facility to manage COVID-19 as well as other common diseases, amongst others (9, 10). A study in Rwanda found that there was a reduction in malaria testing at health facilities during the pandemic but increases in community-based testing (11). In contrast, a study in Uganda found that there was little impact on malaria testing early in the pandemic, but that there was a slight reduction in testing observed as the pandemic progressed (12). Accounting for any changes in testing trends in the context of the COVID-19 pandemic will be required to ensure decision-making on control or elimination strategies are evidence-based (13, 14).

Routine surveillance is an essential intervention to reduce malaria burden, achieve elimination, obtain certification of malaria-free status and prevent re-establishment of transmission (15). When malaria transmission is very low or has been interrupted, any change in care-seeking or testing for malaria could lead to reduced confidence that transmission has been interrupted. Furthermore, any untreated infections in the community can quickly reseed malaria transmission, reversing progress towards elimination. Therefore, the aim of this work was to determine whether there was any pandemic related change in the number of people seeking care or being tested for malaria in Cabo Verde. Any trends observed in the routinely collected surveillance data will be used to determine whether the COVID-19 measures impacted health seeking behavior and testing for malaria in Cabo Verde.

## Methods

### Study setting

**Figure 1:**
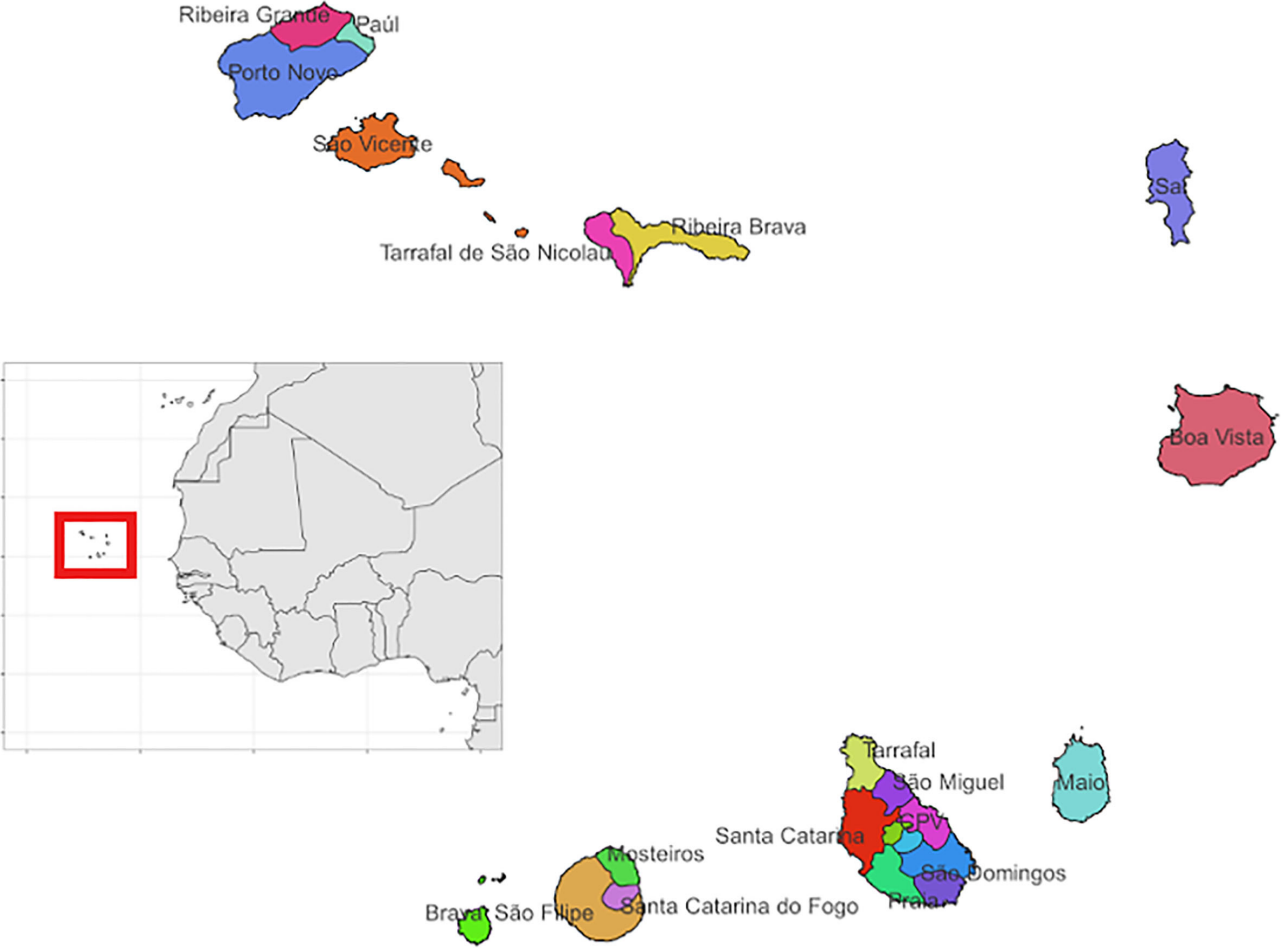
Map showing the municipalities across the islands that make up the archipelago of Cabo Verde. The municipalities are shown using different colours and labelled by the name of the municipality. The inset map highlights its location off the coast of Senegal in West Africa.

Cabo Verde is an archipelago located off the coast of Senegal in West Africa. It is comprised of 9 inhabited islands and has a population of over 550,000 (**Figure 1**) (16). The population is of mixed African and European heritage and almost half of the population resides on Santiago Island, where the capital city Praia, is located (17). Tourism and international shipping are the main economic activities in the country (18). Typical of Sahelian countries, the country is arid with temperatures ranging from a maximum of 25 to 30 degrees Celsius between August and October and lows of 19 to 25 degrees Celsius in January and February. Rainfall in the country is irregular and it experiences periodic droughts with average annual precipitation up to 700 mm in high-altitude areas of the country (19).

The health system in Cabo Verde is coordinated nationally and primarily consists of public institutions. Health services are offered through two reference hospitals, four regional hospitals, 30 health centers, 34 health posts with clinically trained staff as well as 113 Health Units across 22 municipalities (20). Malaria is a notifiable disease. All suspected malaria cases are tested by rapid diagnostic test and microscopy and positive tests are confirmed using molecular methods. Both testing and treatment are available free of charge at public facilities. The last locally acquired malaria case was reported in January 2018 after the country experienced a large outbreak with 428 cases in 2017 (16). *Plasmodium falciparum* was the predominant species when transmission was endemic and is also the parasite most likely to be imported due to frequent travel to the African continent. The high malaria transmission season in Cabo Verde was associated with the rainy season which, is typically between October and December. As of January 2021, the country had experienced three consecutive years of zero indigenous cases of malaria and was eligible to apply for certification of malaria elimination from the World Health Organization (WHO) (21).

On March 10, 2020, the national COVID-19 contingency plan was finalized in Cabo Verde. The first case of COVID-19 was confirmed on March 19, 2020 on the island of Boa Vista and containment measures were quickly implemented. These measures included strengthening surveillance, banning international and domestic air and sea travel, mask wearing, stay at home orders and quarantine measures, amongst others. Island-specific measures were implemented depending on when cases were first identified and case numbers. Measures were gradually lifted as COVID-19 case numbers declined. As part of the response, diagnostic centers were established on 5 of the 9 inhabited islands to ensure access to testing for all 22 municipalities in the country. Vaccination began in on March 19^th^, 2021, one year after the first case was reported 84.6% of the population had received 2 doses as of May 2022 (22).

### Data collection

Routinely collected surveillance data reported by the Ministry of Health was aggregated for this analysis. The number of outpatient visits, malaria tests conducted and positive malaria tests were collected per month and per health facility in Cabo Verde from 2017 through 2020 and combined into a single database. For the reference hospital in Praia from which most malaria cases are reported, individual-level, anonymized patient data for those tested for malaria was available for analysis. This data was summarized by month to ensure a consistent format for data analysis.

Next, the number of COVID-19 tests performed and the number positive tests were extracted from government statistics published online from March through December 2020 (22). Testing was reported at the level of municipality, therefore the database consisted of the number of COVID-19 tests conducted and the number of positive tests per month and per municipality from March through December 2020. Individual-level data on COVID-19 cases including age and gender were not publicly available and therefore could not be included in this analysis. Finally, the timing of COVID-19 measure implementation and cessation was obtained from government reports. The resulting database included the type of measure, whether it was implemented nationally or was specific to a particular island or municipality, the date of implementation and cessation.

This study is a secondary analysis of routinely collected surveillance data available in the public domain. Ethical approvals for this work were obtained from The National Ethics Committee in Cabo Verde (32/2021), London School of Hygiene & Tropical Medicine (26386), and the World Health Organization (0003639).

### Data analysis

The spatial resolution of the outpatient visits and malaria data were aggregated to the municipality level to match that of the available COVID-19 testing data. Exploratory analysis and data visualization were conducted to assess trends in outpatient visits, malaria testing, and COVID-19 testing. Next, an autoregressive integrative moving average (ARIMA) interrupted time series analysis was conducted to assess temporal trends and any changes (23). The time series included data over 48 months from January 2017 through December 2020; January 2017 – February 2020 was considered as the COVID-19 pre-pandemic period and March – December 2020 as the pandemic period. Covariates tested to explain the observed trends included the date that the first COVID-19 case was confirmed in Cabo Verde, COVID-19 testing and positivity rates, as well as the timeline of COVID-19 events. Specific events that were tested to assess the malaria testing and positivity trends included the date the first COVID-19 case was confirmed, when international and national flights were stopped, whether a full or partial lockdown was imposed, mask wearing mandates, when municipal elections took place, and when COVID-19 testing was scaled up (as measured by the number of tests conducted per month). Trends were assessed at both the national and island level. Different transfer functions to model impact were tested including as a step change and a ramp, or a gradual change in the slope (24). The best model fit was ascertained according to the AIC value and confirmed using standard model diagnostic. The best fitting model was used to predict trends in outpatient visits and malaria testing. The percent difference between the predicted and observed trend was calculated. The analysis was conducted using the forecast package and the automated model fitting in R (V 4.1.1).

## Results

**Figure 2:**
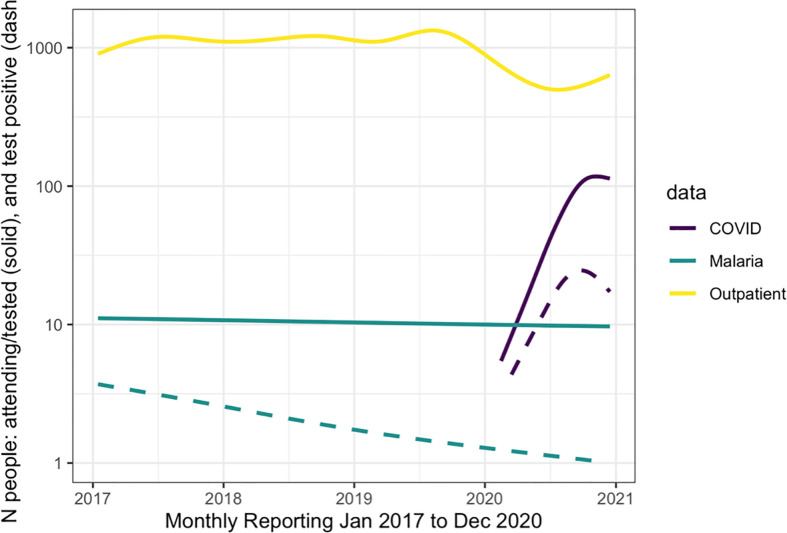
The reported number of people attending the outpatient department (yellow line), for malaria (teal lines) and for COVID-19 (purple line) from January 2017 to December 2020. The solid lines show the number of people tested for each pathogen and the dashed line represents those who tested positive.

**Figure 3:**
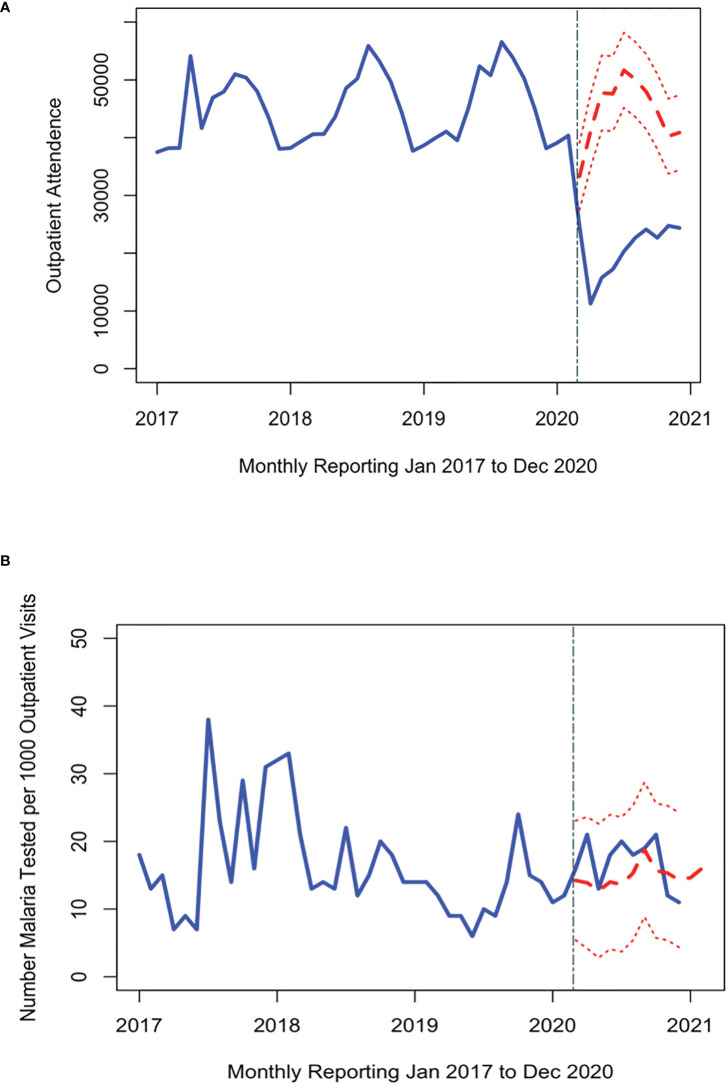
Time series of the number of people attending the outpatient department **(A)** and malaria tests conducted per 1000 outpatient visits **(B)** each month (blue line) from 2017 through 2020. The red dashed line shows the expected trend of number of malaria tests had the pandemic had no impact on malaria testing (red line with 95% credible interval). The first malaria case reported in Cabo Verde is indicated by the grey dashed line.

Over the study period, there were over 1.9 million outpatient visits in Cabo Verde with an average of 543,000 consultations per year, or 2,057 per month during 2017-2019 (**Figure 2**). The number of outpatient visits decreased to a total of 287,236 in 2020 with an average of 1,088 per month (**Table 1**). Of all outpatient visits reported during this period, the majority were in Santiago, followed by São Vicente, and Sal with 22.0%, 13.5% and 11.3% of visits, respectively (**Supplemental Figure 1**). The interrupted time series model for outpatient visits included a 12-month seasonal period with a first order autoregressive term (**Table 2**; **Supplemental Figure 2**). The date the first COVID-19 case was confirmed in the country was the only variable significantly associated with trends in care-seeking. The predicted trends in outpatient visits from the best fitting model suggest that there was a 28% reduction in care-seeking in Cabo Verde after the start of the COVID-19 pandemic (**Figure 3A**). Results were similar when assessing island-specific trends (**Supplemental Figure 4**).

**Table 1:** Summary of the data on routine outpatient visits, malaria and COVID-19 testing and positivity aggregated from routinely collected data from Cabo Verde from January 2017 through December 2020.

	Year			
	2017	2018	2019	2020
Outpatient Visits – Total Annual (Municipality Range of Total Annual Number of Visits)	535,582 (4,022 – 116,284)	542,141 (4,069 – 120,072)	551,188 (4,113 – 121,488)	287,236 (2,199 – 64,199)
Outpatient Visits – Mean Number of Visits Per Month, Per Facility (Municipality Range of Mean Number of Monthly Visits per Facility)	2,029 (335 – 9,690)	2,054 (339 – 10,006)	2,088 (343 – 10,124)	1,088 (183 – 5,350)
Malaria Tests – Total (Municipality Range)	9,733 (0 – 7,160)	10,023 (0 – 8,429)	6,904 (0 – 5,464)	4,380 (0 – 3,811)
Number Positive Malaria Tests (Municipality Range)	446 (0 – 436)	21* (0 – 13)	40 (0 – 20)	10 (0 – 5)
Number COVID-19 Tests (Municipality Range)	–	–	–	26,667 (202 – 3,300)
Number Positive COVID-19 Tests (Municipality Range)	–	–	–	9,180 (24 – 2,943)

*last indigenous case reported in January 2018

**Table 2:** Outputs of best fitting time series models with a 12-month periodicity for outpatient visits (ARIMA (1,0,0) (1,0.0) (12)) adjusting for the date of first COVID-19 diagnosis in the country and malaria testing rates per 1000 outpatient visits, adjusting for COVID-19 testing timeseries in Cabo Verde (ARIMA (1,0,0) (1,0.0) (12)).

	Coefficient	Standard Error	95% Confidence Interval	p-value
OUTPATIENT ATTENDANCE				
Autoregressive Term (1)	0.487	0.158	0.177 - 0.798	0.0021
Seasonal Component (1)	0.524	0.140	0.268 – 0.818	0.0001
Intercept	44574.249	2012.635	40629.56 – 48518.94	<0.0001
Date first COVID-19 Case	-23334.489	2703.888	-28634.01 - -18034.97	<0.0001
MALARIA TESTING				
Autoregressive Term (1)	0.464	0.137	0.198 – 0.733	<0.0001
Seasonal Component (1)	0.359	0.185	-0.005 – 0.722	<0.0001
Intercept	15.81	2.47	10.958 – 20.659	<0.0001
Date first COVID-19 Case	2.23	3.62	-4.866 – 9.333	<0.0001

Malaria testing in Cabo Verde has generally declined since 2017, consistent with the reduction in malaria transmission. During the study period, 31,040 people were tested for malaria across the country, an average of 37, 38, 26, and 16 tests per month in 2017, 2018, 2019, and 2020 respectively (**Figure 2**). Most malaria tests (80.1%) and confirmed malaria infections (91.7%) were reported in Praia, the capital city. The best fitting interrupted time series model for malaria testing rates included a 12-month seasonal period with a first order autoregressive term. The timing of the measures implemented to contain COVID-19 were not associated with trends in malaria testing (**Table 2**, **Supplemental Figure 3**). The predicted trends in malaria testing rates based on the best fitting model suggest there was a non-significant 8% increase in malaria testing rates per 1000 outpatient visits after the first case of COVID-19 was confirmed in the country (**Figure 3B**). Results were similar when assessing the island-specific trends (**Supplemental Figure 4**).

## Discussion

Cabo Verde has made substantial gains in reducing malaria burden over the past decade and is poised to achieve certification of malaria elimination. Despite the advances made in recent years the COVID-19 pandemic posed enormous challenges to the consolidation of these gains. With this secondary analysis of routinely collected surveillance data, we show that despite a significant reduction in care-seeking after the first case of COVID-19 was confirmed in Cabo Verde, a reduction that was sustained through 2020, there was minimal impact on malaria testing. We also show that malaria testing rates did not change despite a reduction in care-seeking, suggesting that the sensitivity of the surveillance system to detect malaria in Cabo Verde remained high, despite the impact of the pandemic on the health system (25).

This study includes routinely collected surveillance data for the first nine months of the COVID-19 pandemic in Cabo Verde. It is possible that the reduction in care-seeking and maintenance of malaria testing was short term and that it may have rebounded to pre-pandemic levels as restrictions were lifted, vaccination rates increased, or as perceptions of COVID-19 risk evolved in the population (26, 27). Continuous monitoring of the impacts of COVID-19 on malaria surveillance, as well as for other illnesses, will be important to support decision-making. The potential impact on disease surveillance is particularly acute for diseases that exhibit similar symptoms and are also endemic (28). Teasing apart the true disease trends, independent of any measurement bias associated with the pandemic, will enable changes in transmission to be quickly detected and measures to be adapted accordingly (29). Similarly, identifying when surveillance trends return to pre-pandemic levels would ensure that an appropriate baseline for comparison can be defined.

A strong surveillance system is essential when confirming that a disease has been eliminated (30). Malaria testing in Cabo Verde declined over the study period, particularly since 2017 when the last malaria outbreak was recorded. One prerequisite for supporting malaria elimination is achieved and can be sustained is ensuring that potentially infected individuals have access to timely testing and if positive, treatment with effective antimalarial drugs. Any change in testing raises questions about access to testing for those who require it and a change in care-seeking could raise doubts regarding whether potentially infected individuals are seeking care. If true, the risk of disease introduction and subsequent local transmission going undetected increases and introduces doubt as to whether elimination has been achieved. That malaria testing rates remained relatively stable during the acute phase of the COVID-19 pandemic suggests that the surveillance system in Cabo Verde remained vigilant against malaria despite the COVID-19 pandemic related strain on the health system.

This study is subject to the following limitations. This study was a secondary analysis of routinely collected surveillance data and our ability to assess more granular trends was limited by the relative paucity of available data. Knowing the age and sex of malaria and COVID-19 cases would help identify whether there were demographic shifts in testing and spatial information on patient residence could inform whether all populations were represented in the surveillance data. Similarly, there are multiple factors that influence care-seeking decisions including distance to a health facility. Yet, factors impacting care-seeking are expected to have been relevant in both the pre-pandemic and pandemic period. Although we cannot exclude changes in other elements that would contribute to the change in care-seeking during the pandemic period, we expect any impact to be non-differential. Assessing changes in trends for other drivers of care-seeking (e.g., maternal and child health visits, vaccinations, etc.) would be helpful to validate the trends observed for malaria and assess the full scale of the impact of the pandemic on public health surveillance in general. However, this data was not available for this analysis. Finally, a longer time series of data since the start of the pandemic would enable assessments of longer-term trends or changes in the surveillance data but was not available within the timeframe of this analysis.

Ultimately, that malaria testing was maintained during the first 9 months of the COVID-19 pandemic in Cabo Verde is reassuring. Understanding these trends in other settings will be crucial to ensure that malaria surveillance data for this period can be interpreted appropriately. Furthermore, prospectively following trends in malaria positivity or care-seeking would be informative and help identify if and when surveillance trends return to pre-pandemic levels. Similarly, identifying if or how surveillance trends change through the different waves of the pandemic could help inform the optimal strategies when preparing for the next pandemic. For surveillance to be an effective intervention, the data informing decision-making must be robust and reflect the true disease burden in the population.

## Data availability statement

The original contributions presented in the study are included in the article/**Supplementary Material**. Further inquiries can be directed to the corresponding author.

## Ethics statement 

Ethical approvals for this work were obtained from The National Ethics Committee in Cabo Verde (32/2021), London School of Hygiene & Tropical Medicine (26386), and the World Health Organization (0003639).

## Author contributions

AT, GS, AD conceived the study. AD, HB collected and aggregated the routine surveillance data for both malaria and COVID-19. GS, HB conducted the data analysis and GS wrote the paper. All authors provided critical input and approved this manuscript.

## Funding

GS received salary support through her Henry Wellcome postdoctoral fellowship from the Wellcome Trust [No: 204693/Z/16/Z].

## Acknowledgments

We would like to acknowledge the National Malaria Team in Cabo Verde for making the data available, program staff at the local level diligently reporting the data, and Prof. Chris Drakeley for providing constructive input on the results of this research.

## Conflict of interest

The authors declare that the research was conducted in the absence of any commercial or financial relationships that could be construed as a potential conflict of interest.

## Publisher’s note

All claims expressed in this article are solely those of the authors and do not necessarily represent those of their affiliated organizations, or those of the publisher, the editors and the reviewers. Any product that may be evaluated in this article, or claim that may be made by its manufacturer, is not guaranteed or endorsed by the publisher.
