# Supplementary material for: Sustaining surveillance as an intervention during the COVID-19 pandemic in Cabo Verde and implications for malaria elimination
Source: Front Immunol. 2022 Oct 6;13:956864. doi: 10.3389/fimmu.2022.956864 (PMC9582766; doi:10.3389/fimmu.2022.956864)
Supplement: Supplementary file 1 [file DataSheet_1.docx]

**Supplementary Figures**

**Suppl Figure 1:** The reported number of people attending the outpatient department (yellow line), for malaria (teal lines) and for COVID-19 (purple line) from January 2017 to December 2020. The solid lines show the number of people tested for each pathogen and the dashed line represents those who tested positive. Trends are shown for each of the nine inhabited islands.


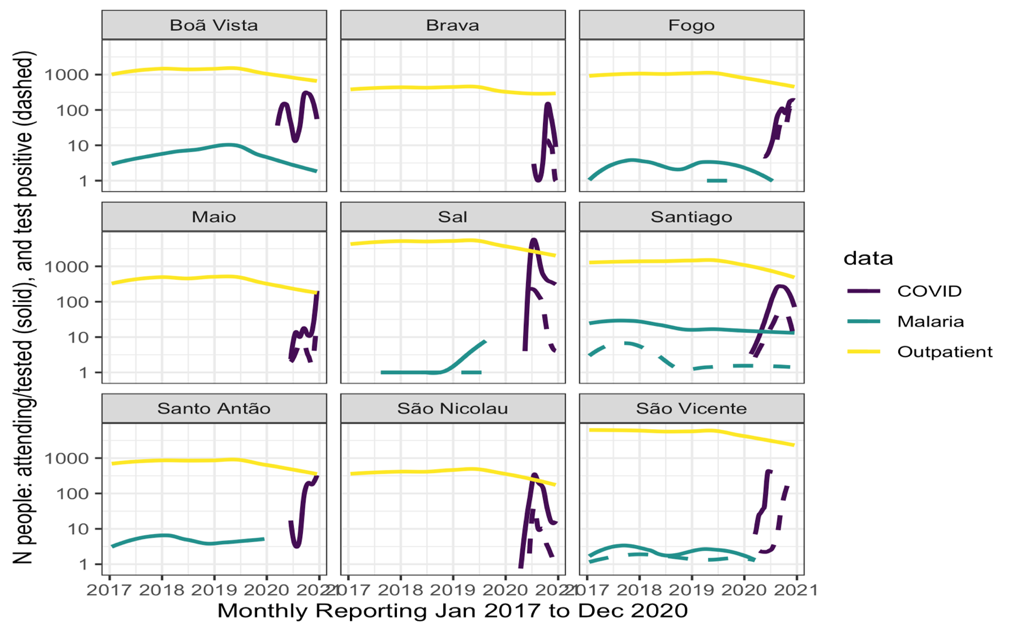


**Suppl Figure 2:** Residual diagnostic plots of ARIMA assessing outpatient trends, adjusting for date of first COVID-19 diagnosis


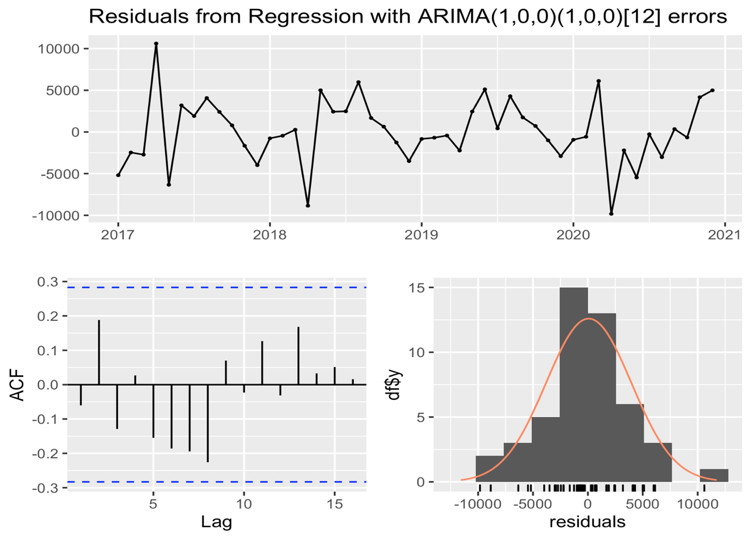


**Suppl Figure 3:** Residual diagnostic plots of ARIMA assessing trends in malaria testing rates per 1000 outpatient attendees, adjusting for time series of number of COVID-19 tests conducted per month.


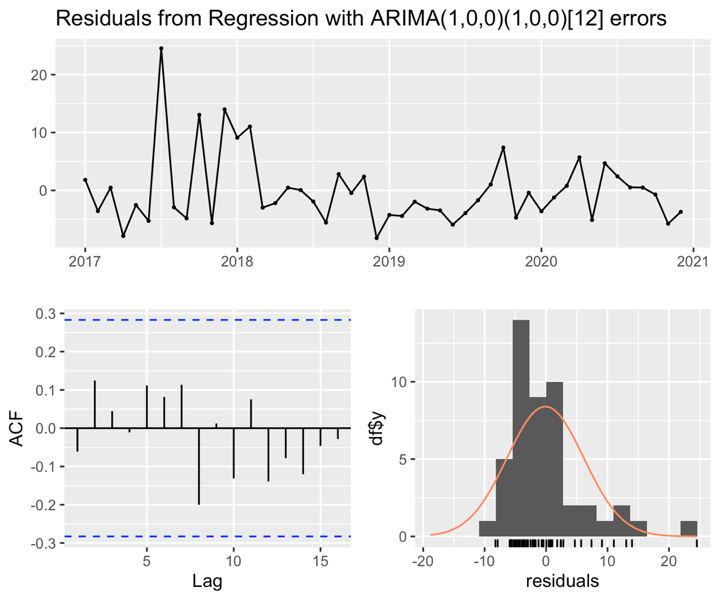


**Suppl Figure 4:** Results of timeseries analysis for trends in outpatient attendance and malaria testing (Columns) per island (Rows) over the four-year study period. The blue lines highlight the observed trend based on the routine data collected by the health facilities with the red dashed line, and corresponding 95% confidence bands (red dotted line), representing the predicted trend based on the time series after the start of the COVID-19 pandemic (grey vertical line).

| **Island** | **Outpatient** | **Malaria** |
| --- | --- | --- |
| Boa Vista |  |  |
| Brava |  | No Malaria Tests |
| Fogo |  |  |
| Maio |  |  |
| Sal |  |  |
| Santiago |  |  |
| Santo Antão |  |  |
| São Nicolau |  | No Malaria Tests |
| São Vicente |  |  |
